# Early visual experience influences haptic cross-sectioning ability

**DOI:** 10.1007/s00426-026-02310-9

**Published:** 2026-05-22

**Authors:** Monica Gori, Margherita Di Gaudio, Diego Torazza, Silvia Zanchi

**Affiliations:** 1 Unit of Visually Impaired People, Istituto Italiano di Tecnologia, Genoa, Italy; 2 Institute for Human & Machine Cognition, IHMC, 40 South Alcaniz St, Pensacola, FL 32502 USA; 3 DIBRIS Department, Università di Genova, Genoa, Italy; 4 Generative Bionics S.R.L, Genoa, Italy

## Abstract

**Introduction:**

Cross-sectioning task requires participants to identify the two-dimensional (2D) internal shape of cross-sectioned three-dimensional (3D) solids. This task demands advanced spatial reasoning abilities, including the mental manipulation of both two-dimensional and three-dimensional objects. While previous research has highlighted the importance of vision in understanding objects’ spatial features, the relationship between successful cross-sectioning and visual impairments remains unclear.

**Methods:**

The present study aims to investigate how early-life vision guides the ability to construct and transform spatial representations through touch, using a haptic 3D-printed version of the Santa Barbara Solid Test. Our sample consisted of four groups of participants: early blind (*N* = 11), late blind (*N* = 8, onset of blindness: after 6 years old), low vision (*N* = 5, vision acuity between 3/10 and 1/20), and blindfolded sighted (*N* = 13). All participants were asked to haptically explore and recognize cross-sections of 3D-printed solids of varying difficulty levels (*N* = 10 trials) and to identify the associated correct section among four options. We analyzed performance using a generalized linear mixed model, with accuracy (correct versus incorrect) as the outcome and group and difficulty as predictors.

**Results:**

The group (i.e., onset of blindness) emerged as the only significant predictor for task performance (*p-value* < 0.001). In particular, early blind participants show lower probability of correctly identifying cross-section of solids compared to the other groups (all *p-values* ≤ 0.02).

**Discussion:**

These findings show that visual deprivation in the first years of life impacts the development of effective haptic spatial skills, highlighting the need for tailored rehabilitation programs, particularly in the context of multisensory spatial reasoning.

**Supplementary Information:**

The online version contains supplementary material available at 10.1007/s00426-026-02310-9.

## Introduction

Spatial abilities, defined as a “skill in representing, transforming, generating, and recalling symbolic, nonlinguistic information” (Linn & Petersen, 1985, p. 1482), are crucial for daily life, encompassing a wide range of processes and skills, including object manipulation and use. One specific task that tests these abilities is cross-sectioning, in which participants are required to infer and understand the spatial features of a three-dimensional (3D) object by imagining its internal two-dimensional (2D) features (Carroll, 1993; Cohen & Hegarty, 2007, 2012). A cross-section is the resulting plane or surface after a 3D object is cut using a 2D plane. This task is essential not only in specialized domains such as human anatomy (Hegarty et al., 2009; Langlois et al., 2017) or the engineering field (e.g., Gerson et al., 2001), but also in everyday activities, for instance, assembling furniture based on visual instructions or slicing food at specific angles. Successful cross-sectioning requires a robust spatial representation of objects and the ability to mentally manipulate both 2D and 3D forms. Importantly, cross-section is not a unitary and homogenous ability. We describe cross-sectioning different processing stages as follows: (i) forming a coherent representation of the 3D object; (ii) transforming this representation, considering the relationship between the section and the solid, inferring the resulting cross-sectional shape; (iii) maintaining it in memory. Relative to stage ii), efficient orientation discrimination abilities are necessary to comprehend the direction of the cutting plane relative to the object. Successful cross-sectioning thus requires maintaining orientation through tactile cues. Given its reliance on spatial reasoning and orientation discrimination, cross-sectioning is a unique task for studying sensory processes in object manipulation and spatial processing across both two- and three-dimensional spaces.

Vision is the dominant sense for conveying spatial information (Alais & Burr, 2004), enabling a clear understanding of object orientation, the spatial relationships between environmental elements, and the formation of an allocentric perspective. When vision is absent, spatial understanding is significantly affected. While in simple tasks, such as stimulus localization, vision is not strictly needed for spatial calibration (e.g., Lessard et al., 1998), in spatially challenging activities, visually impaired people show impaired performance if compared with their sighted controls (Gori et al., 2010, 2014). Previous research demonstrates that the occipital cortex is recruited for tactile object recognition in the congenitally blind population (Amedi et al., 2010), and this network is shared with sighted individuals (Xu et al., 2023). This suggests that even in the absence of vision, regions traditionally associated with visual processing continue to play a fundamental role in spatial tasks, highlighting the cross-modal utility of these brain areas. Loss of vision early in life results in greater reliance on touch for object identification (Occelli et al., 2016). Touch can indeed provide positional and spatial relationship information, and spatial abilities assessed in visual and tactile modalities correlate (Langlois et al., 2024). Haptic perception, which involves interacting with objects through active touch (Grunwald, 2008; Lederman & Klatzky, 1987, 2009; Heller & Ballesteros, 2006), is key to this process. Besides, a greater reliance on haptic exploration in blindness does not necessarily translate to an enhanced ability to comprehend objects through touch alone. Prior studies show great variability in haptic abilities among the blind population (Alary et al., 2009; Amedi et al., 2010; Burton et al., 2004; Grant et al., 2000; Voss et al., 2016), likely indicating that the performance in spatial representation and processing may be task-related. Indeed, previous findings have shown that people with visual disabilities behave similarly or even outperform sighted controls in simple haptic tasks, involving texture and frequency discrimination, and tactile time perception (Alary et al., 2009; Morash et al., 2012; Norman & Bartholomew, 2011; Röder et al., 2004). Besides, prior literature demonstrated that congenitally blind people performed worse in haptic scene recognition, when complex change of perspective is required (Pasqualotto & Newell, 2007). In addition, haptic orientation task is strongly affected in the absence of early vision (Gori et al., 2010). Therefore, if vision plays a crucial role in calibrating other sensory modalities for spatial perception (Gori, 2015), early visual deprivation may differentially affect specific stages of cross-section reasoning. We would expect individuals with early vision loss to experience impairments in spatial tasks that require complex processing of spatial features in objects. Indeed, even with strong experience with everyday objects (i.e., tools) congenitally blind people show high variability and biases in the precise representation of objects, with vision contributing to calibrating object configurations (Tian et al., 2024). Conversely, it is plausible that individuals who lost vision later in life or are affected with low vision might benefit from prior visual calibration and extensive experience with haptic exploration, particularly if their blindness has not persisted for an extended period. In this sense, cross-calibration theory (Gori, 2015) could explain the controversial findings in the mentioned previous studies (Alary et al., 2009; Amedi et al., 2010; Burton et al., 2004; Grant et al., 2000; Morash et al., 2012; Norman & Bartholomew, 2011; Röder et al., 2004; Voss et al., 2016), suggesting that the congenitally blind group should respond poorly when the task requires refined spatial cognition.

The aim of the present study is to unveil how early-life vision shapes the ability to construct and transform spatial representations through touch. To address this aim, we examined performance on a haptic cross-sectioning task, which requires participants to infer internal 2D features of 3D objects and maintain orientation during exploration. This ability is tested across different visually impaired populations, including low vision, late blind, early blind, and sighted controls. For this purpose, we created a 3D-printed version of the Santa Barbara Solid’s Test (SBST) that includes real 3D shapes with actual planes that intersect them (Gori et al., 2024). This task is designed to assess the participants’ ability to mentally visualize and comprehend cross-sections of objects. Young children showed improved geometrical comprehension when exposed to visuo-haptic solids than visual ones alone (Gori et al., 2024), suggesting that tactile exploration provides valuable information for cross-sectioning comprehension.

**Fig. 1:**
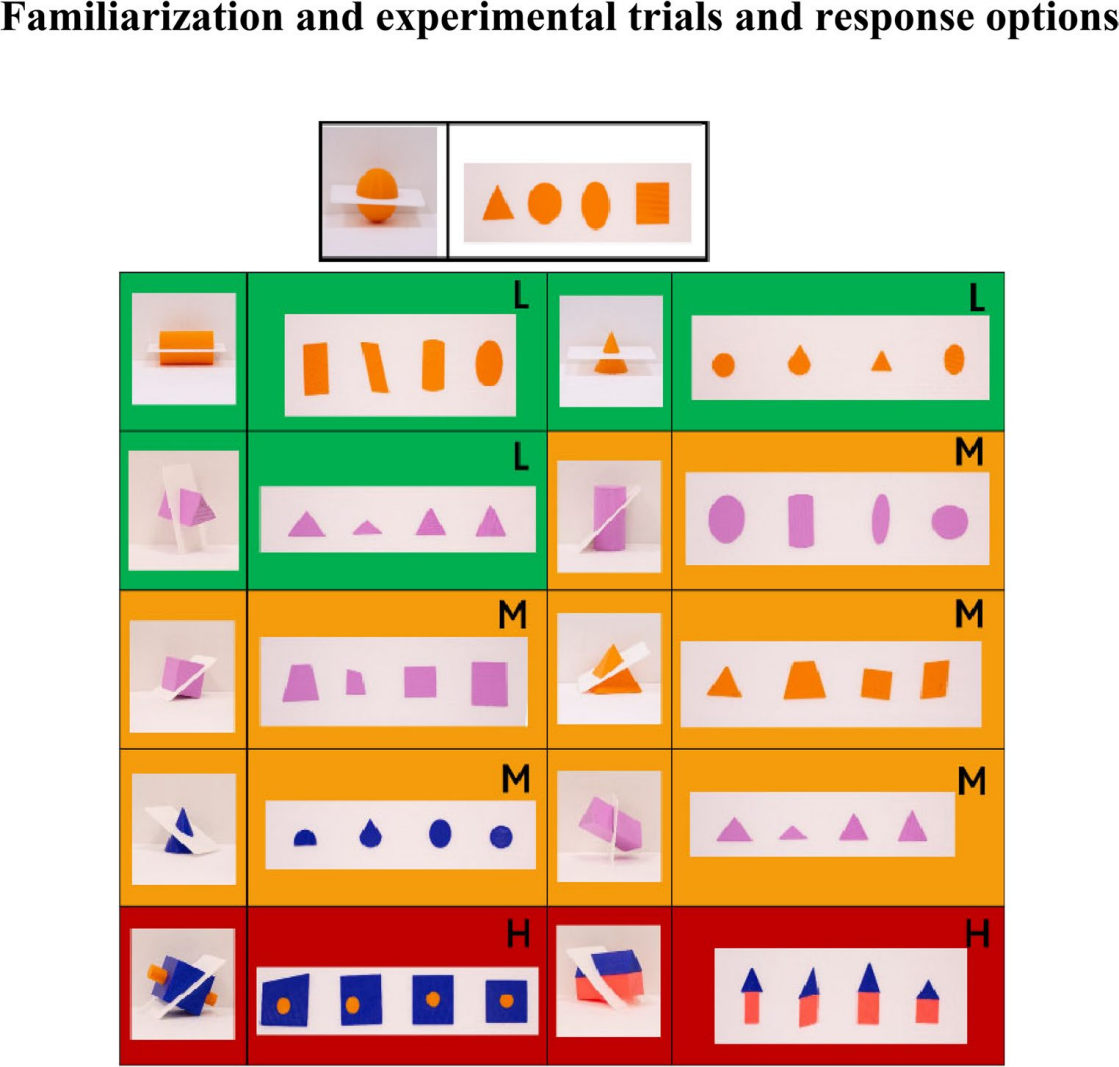
Familiarization trial (at the top) and the ten experimental trials. To each 3D solid, four response options are associated on the right. Background colors and letters refer to the level of associated difficulty (green background, L = low, simple figure and orthogonal cutting plane; yellow background, M = medium, simple figure and oblique cutting plane; red background, H = high, embedded and joined figures with oblique cutting plane)

As mentioned, the task involves different processing stages, including forming a coherent representation of the 3D object, transforming this representation, and maintaining it in memory. We manipulated task difficulty by varying the type of solid and the orientation of the cutting plane, similarly to the visual version of the task. Specifically, we selected simple solids with orthogonal (the cut is perpendicular to one of the principal axes of the object) and oblique (the cut is at an angle that is not perpendicular to any principal axis) sections, on simple, embedded and joined solids (Fig. 1). We employed simple geometric solids to minimize the influence of object familiarity on this task. The selected different orientations of cuts were meant to subset different levels of difficulty of spatial reasoning. In the visual SBST, orthogonal sections primarily require accurate shape construction and basic alignment processes. Besides, embedded and joined solids are thought to require more effort in mentally maintaining their spatial configuration, and objects with oblique cutting planes involve more difficult object transformation (Cohen & Hegarty, 2007). Thus, with these different levels of difficulty we have the specific objective to explore whether performance differences emerge in conditions that place greater demands on spatial transformation of the objects (oblique cuts) or in configurational maintenance (embedded or joined solids).

Based on this framework, we formulated two hypotheses. First, we hypothesize that early visual loss may primarily affect orientation discrimination and spatial transformation processes, which are known to rely strongly on visual calibration (Gori et al., 2010). As second hypothesis, we posit that task variations will reveal differential abilities across the different groups, particularly in terms of tactile exploration proficiency, merging haptic object exploration and orientation perception.

## Method

### Participants

We tested blindfolded sighted controls and visually impaired participants with the same experimental procedure. Visually impaired individuals were recruited from a large internal database maintained by the authors’ Institute. Details of the ages, sex and visual impairments of participants are displayed in Table 1 and reported in the Results section. Unfortunately, some information is missing for one early blind participant (specific pathology and visual residual) and the low vision participants (sex and specific onset of their visual impairment); this limitation, along with the small sample size of each group due to the challenges of recruiting clinical populations, is addressed further in the Limitations section. The local ethics committee approved the study. All participants either read themselves or had them read aloud, and signed written informed consent forms under the Declaration of Helsinki.

**Table 1:** Clinical details of visually impaired participants

Sex	Age	Visual impairment	Onset of Blindness	Age of complete blindness	Pathology	Visual residual
F	32	Blind	Early	Birth	Retinitis pigmentosa + myopia	1/20, visual field < 1%, lights, shadows, shapes during day light, nothing at night
F	31	Blind	Early	Before birth	retinopathy of prematurity	no vision
F	33	Blind	Early	Before birth	retinopathy of prematurity	no vision
M	28	Blind	Early	Birth	Leber congenital amaurosis	no vision
M	48	Blind	Early	Before birth	Congenital optic nerve atrophy	no vision
M	23	Blind	Early	-	-	-
F	21	Blind	Early	-	retinopathy of prematurity	lights and shadows
M	55	Blind	Early	-	retinopathy of prematurity	lights and shadows
F	43	Blind	Early	Before birth	Microphthalmos and congenital cataract	no vision
M	53	Blind	Early	-	unknown	lights and shadows
M	44	Blind	Early	Birth	retinopathy of prematurity	no vision
F	31	Blind	Late	6	Optic nerve tumor	lights and shadows
M	61	Blind	Late	11	Uveitis	lights and shadows
M	30	Blind	Late	19–20	Leber amaurosis	lights and shadows
F	61	Blind	Late	40	retinopathy pigmentosa	lights and shadows
M	34	Blind	Late	17	Corneal opacity	No vision
M	34	Blind	Late	20	Degenerative oculopathy in both eyes	lights and shadows, visual field < 1%
F	44	Blind	Late	18	Accident, lost retina	lights and shadows
M	51	Blind	Late	40	optic nerve subatrophy due to benign tumor	R Eye: lights and shadows, L Eye: < 2/10. Both eyes: No visual field, nystagmus
F	44	Low vision	-	-	-	Residual between 3/10 − 1/20
-	33	Low vision	-	-	-	Residual between 3/10 − 1/20
F	79	Low vision	-	-	-	Residual between 3/10 − 1/20
-	59	Low vision	-	-	-	Residual between 3/10 − 1/20
-	73	Low vision	-	-	-	Residual between 3/10 − 1/20

### Apparatus and stimuli

In this study, we selected 10 items from the Santa Barbara Solids Test, SBST (Cohen & Hegarty, 2007; see the original test at: https://hegarty-lab.psych.ucsb.edu/node/231). We presented all items in 3D-printed solid shapes (material: Polylactic acid, PLA) with a base edge length or diameter of 10 cm; we also 3D printed the cutting plane and the resulting sections used as answer choices (Fig. 1, experimental sections; additional pictures of the solids are provided in Supplementary Information, Figure S1). Importantly, although these flat response options are physically thin 3D objects that can be explored haptically, from here on, we refer to them as “2D sections” to highlight their geometric definition as planar cross-sections and to mirror the visual version of the task. The 3D printing was performed using a Wasp Delta 2040 Turbo2 printer (Wasp S.r.l., Italy). The 3D models were designed using PTC Creo 7.0 CAD software (https://www.ptc.com/en/products/creo), while the print files were generated using Simplify3D^®^ software (https://www.simplify3d.com/). The particularity of our 3D items was the possibility of assembling and disassembling the solid to see how the cutting plane intersects the solid shape. We chose one simple solid (i.e., primitive geometric solid, a sphere) with an orthogonal cutting plane as a familiarization training trial (Fig. 1). As experimental trials, we selected three simple solids with an orthogonal cutting plane, five simple solids with an oblique cutting plane, one joined solid (i.e., figure where two simple solids attached at their edges) with an oblique cutting plane, and one embedded solid (i.e., figure where a single simple solid shape is encased or enclosed within another shape) with an oblique cutting plane. All the shapes directly derive from the ones chosen in the visual version of the SBST (Cohen & Hegarty, 2007). We then classified the trials according to the different levels of difficulty that are reported in the visual version of the task (Cohen & Hegarty, 2007). Specifically, a solid cut by orthogonal plane was considered low difficulty; a solid cut with an oblique plane was labeled medium difficulty; embedded and joined solids with oblique cutting plane were encoded as a high level of difficulty. This classification directly derives from the original visual version of the test.

### Procedure

In the present study, we aimed to evaluate whether visual experience and development influenced the ability to mentally reconstruct the 2D section of haptically explored 3D shapes. Participants were presented with one sample item (a sphere with an orthogonal cutting plane) and ten experimental items, as described in the previous section. The order of items was the same for all participants. Notably, no participant was visually exposed to the stimuli: sighted and visually impaired participants with visual residual either had eyes closed or were blindfolded before entering the experimental room until the end of the whole experiment. To familiarize the participants with the stimuli, the experimenter allowed them to haptically explore the sphere item with their hands. Subsequently, four answer options were given to participants, one at a time, in the order displayed in Fig. 1, from left to right, from top panels to bottom panels. If participants provided the correct answer to the training trial, the experiment could start. Similar to the training trial, participants received each of the 3D shapes, followed by a sequence of four answer options after haptic exploration. Verbally, they indicated whether the correct 2D section was the first, the second, the third, or the fourth. Importantly, no time constraints were imposed for either the haptic exploration of the experimental items or the answer options. The experimenter (M.G.) recorded participants’ responses on a paper sheet featuring the pictures of all the items. Exploration and response times were not recorded.

### Data analysis

For each experimental trial, we considered participants’ responses as correct when they selected the right 2D section among the four options (right answer coded = 1) and wrong if they selected one of the three distracting shapes (wrong answer coded = 0). No trials were excluded. Considering the dichotomic nature of the measured accuracy (correct, incorrect; 1/0), we fitted a generalized linear mixed model (GLMM) considering accuracy as the outcome and group (four levels: sighted SC, low vision LV, late blind LB, early blind EB) and difficulty (low, medium, high) as predictors, taking into account interindividual variability:

$$\begin{array}{c}\mathrm{Accuracy}\;\left(1/0\right)\sim\mathrm{group}\;\left(\mathrm{EB}/\mathrm{LB}/\mathrm{LV}/\mathrm{SC}\right)\ast\\\mathrm{difficulty}\;\left(\mathrm{low}/\mathrm{medium}/\mathrm{high}\right)+\left(1\left|\mathrm{participant}\right.\right)\end{array}$$

Before this simple model, we initially explored a more complex generalized linear mixed model including random slopes for difficulty within participants and random intercepts for participants and items, as recommended in the literature (Barr et al., 2013; Bates et al., 2014). However, this model failed to converge, likely due to the complexity of the random-effects structure relative to the available data. Non-convergence indicates unreliable parameter estimates, so we retained the simpler model with random intercepts for participants only (Barr et al., 2013). The abovementioned final model includes all theoretically relevant fixed effects and accounts for repeated measures, ensuring stable estimation.

All the statistical analyses were computed using R software (version 2023.06.0, PBC, Boston, MA, USA). Specifically, we used g*lmer* function from *lm4e* package (Bates et al., 2014), *emmeans* from *emmeans* package (Lenth, 2025). The GLMM predictors were evaluated using Type III Wald chi-squared tests as implemented in the *Anova* from *car* package (Fox & Weisberg, 2019). To evaluate the sensitivity of our study to detect group differences, we conducted a simulation-based power analysis using the *simr* package (Green & Macleod, 2016). The fitted GLMM served as the data-generating model. We focused on the overall main effect of the group. One hundred simulated datasets were generated to estimate the probability of detecting the observed group effect at α = 0.05.

## Results

**Fig. 2:**
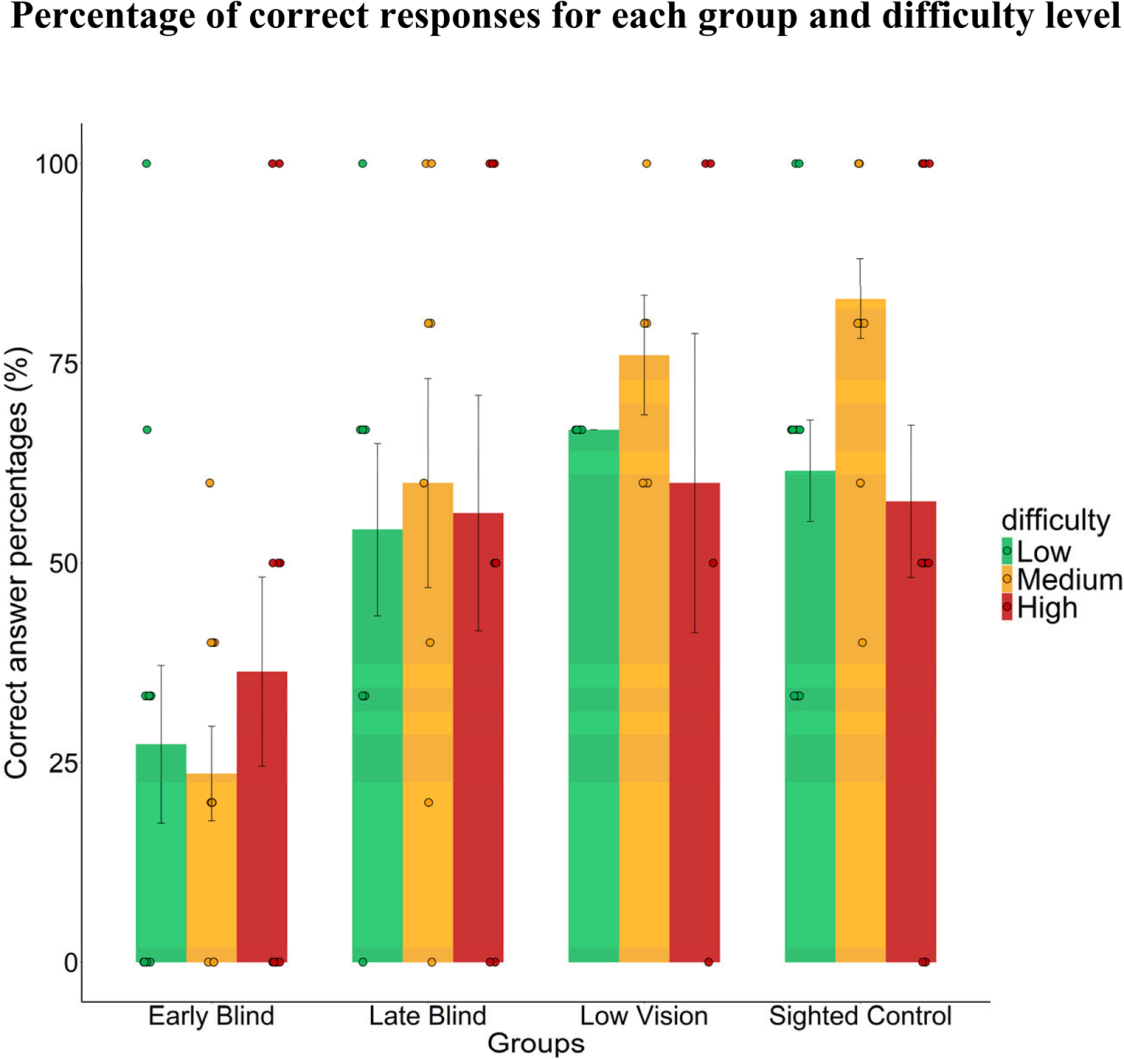
Percentage of correct responses for each group and level of difficulty are shown for illustrative purposes. Early blind group performed significantly worse than other groups. Difficulty level show no specific impact on performance. Each individual point represents the average performance of participants across trials. Bars represent the mean percentage for each group and difficulty level, the error bars represent standard errors

**Fig. 3:**
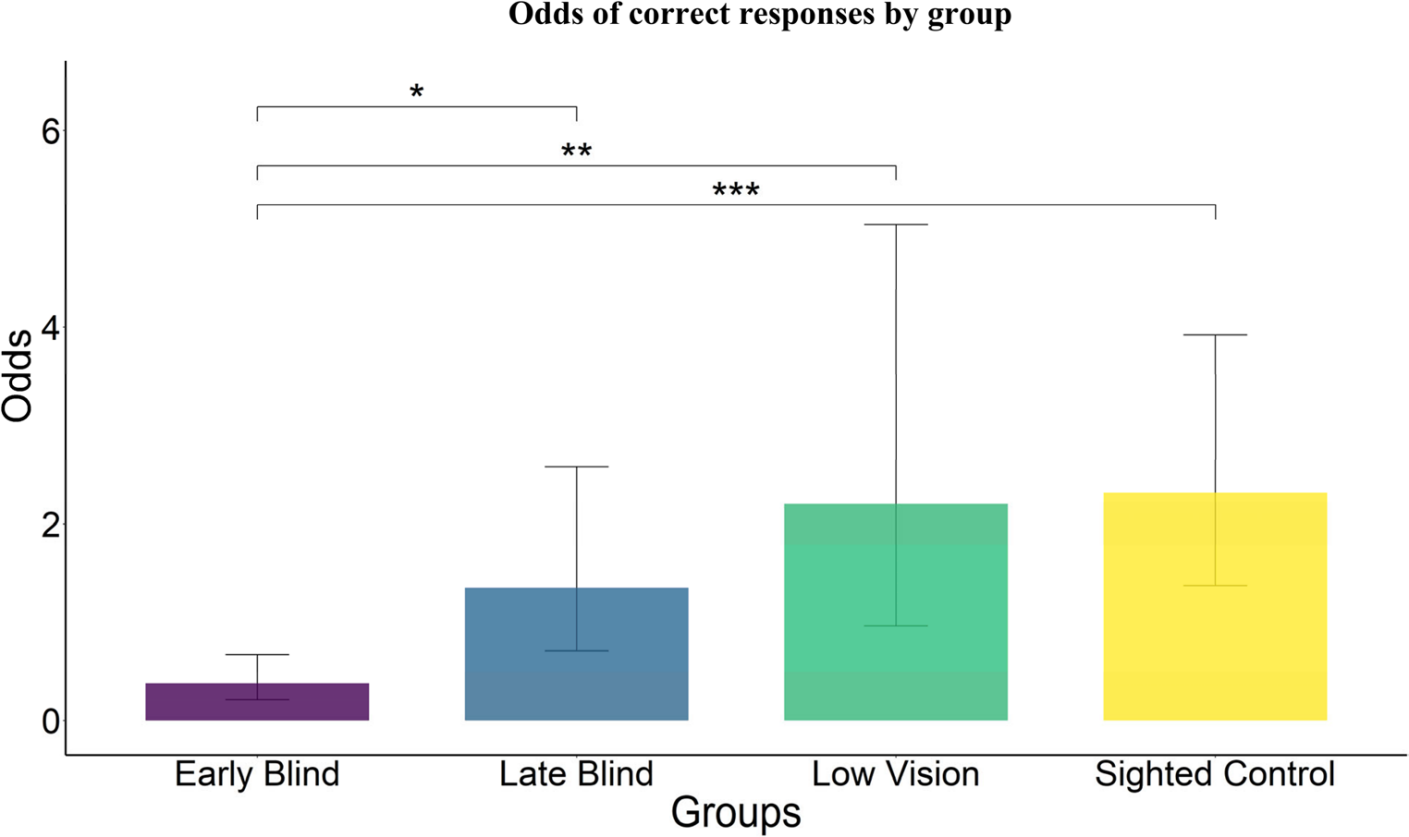
Odds of correct responses for each group. The early blind group performed significantly worse than all the others, which did not differ from each other. Bars represent the estimated odds of success derived from estimated marginal means of the generalized linear mixed model, and error bars indicate the lower and upper bounds of the model-estimated odds (95% confidence intervals). * = *p*-value < 0.05; ** = *p*-value <0.01; *** = *p*-value < 0.001

Sex and ages and other details of the visual impairments of participants are displayed in Table 1. Details of the participants in each group are as follows: blindfolded sighted controls (sample size *N* = 13, 9 Female (69.2%) sighted controls SC; mean age (standard deviation SD) = 31 (5.1) years old) and visually impaired participants (*N* = 11 early blind EB, 5 Female (45.5%), mean age (SD) = 37.4 (11.8) years old; *N* = 8 late blind LB, 3 Female (37.5%), mean age (SD) = 43.2 (13) years old; *N* = 5 low vision LV, at least 2 Female (at least 40%), mean age (SD) = 57.6 (19.3) years old). Every participant responded correctly to the familiarization trial. Internal consistency of the 10 experimental trials accuracy scores was acceptable, with Cronbach’s alpha (KR-20) = 0.76, 95% Confidence Interval CI [0.65, 0.87], across all participants. The percentage of correct responses for each group and difficulty level is depicted in Fig. 2. Regarding the GLMM, as revealed by the Wald chi-squared test, the fixed effect of group was significant (*χ*^*2*^(3) = 23.5; *p-value* < 0.001), while difficulty (*χ*^*2*^(2) = 2.8; *p-value* = 0.24) and interaction were not (*χ*^*2*^(6) = 8.19; *p-value* = 0.22). As shown in Fig. 3, contrasts using *emmeans* function with Bonferroni correction specifically showed that the congenitally blind group (EB = reference level) exhibited a significantly lower performance than the other three groups (versus (vs.) LB: odds ratio = 0.28, *p-value* = 0.02; vs. LV: odds ratio = 0.17, *p-value* = 0.004; vs. SC: odds ratio = 0.16, *p-value* < 0.001), which did not differ from each other (all *p-values* > 0.20). To clarify, odds ratios are the ratio between odds in each group (e.g., SC group is 2.32 times more likely to succeed than to fail (odd = 2.32), while EB group reports odd = 0.38, suggesting more failures than successes: the odds ratio is the ratio between the two, 0.38/2.32 = 0.16).

Despite the modest sample size (*N* = 37 participants in total), the simulation-based sensitivity analysis using the fitted GLMM (*simr* package, 100 simulations) suggested that, under the given conditions, the group effect was detected with high confidence (~99% power, 95% CI: 95–100). This indicates that the detected group differences are likely real, whereas non-significant effects of difficulty and interaction may reflect limited sensitivity to smaller effects. Collectively, these results provide strong evidence for a group effect on accuracy, while acknowledging the need for larger samples to precisely estimate smaller or more nuanced effects.

**Fig. 4:**
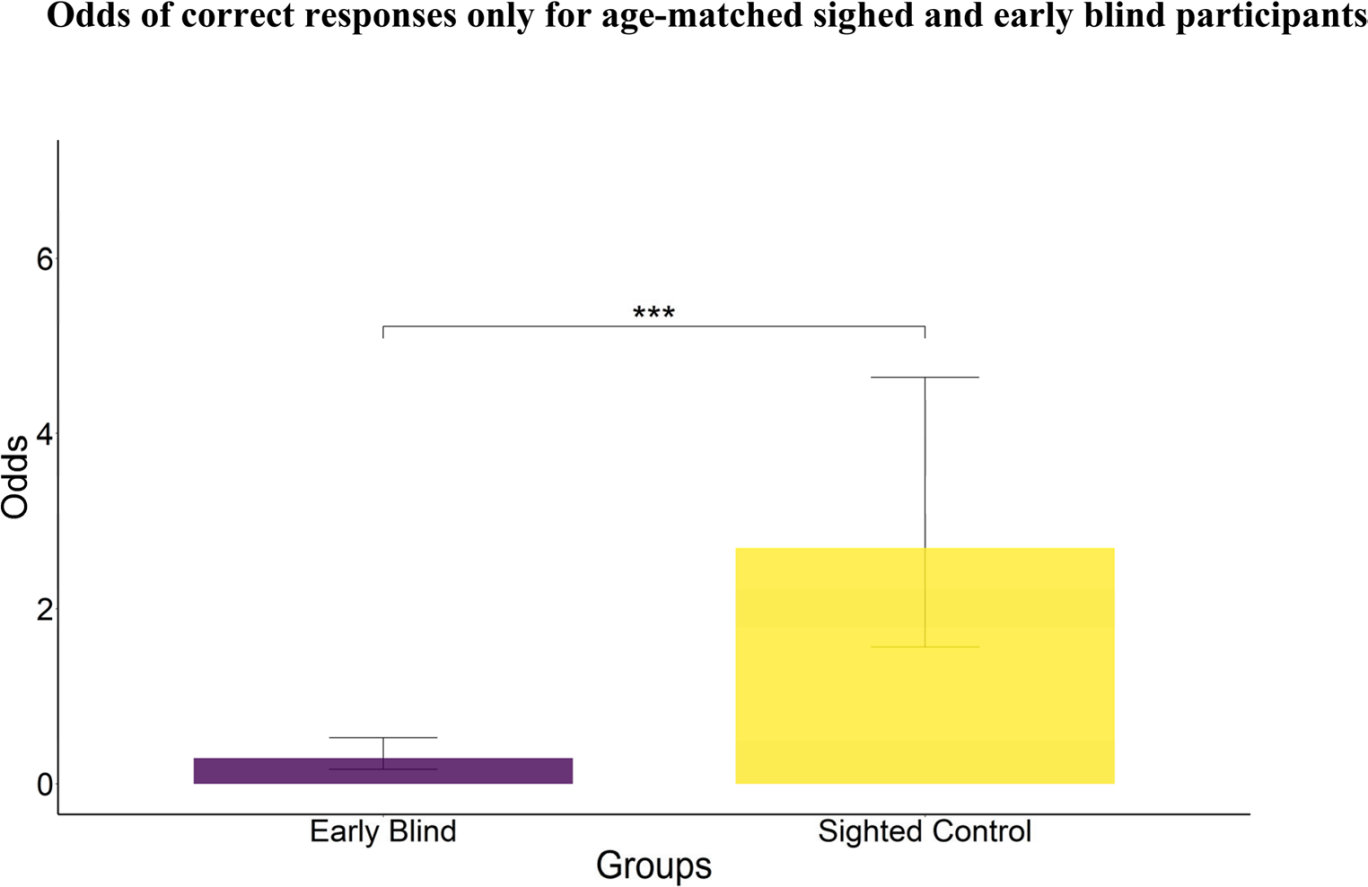
Odds for age-matched early blind and sighted control group. The early blind group performed significantly worse than sighted. Bars represent the estimated odds of success derived from estimated marginal means of the generalized linear mixed model, and error bars indicate the lower and upper bounds of the model-estimated odds (95% confidence intervals).*** = *p*-value < 0.001

Aging has been shown to affect spatial abilities (Techentin et al., 2014). Considering the heterogeneity of blind participants, it was challenging to select exclusively age-matched participants in all groups. To further control the potential confounding effect of age, we selected a subset of sighted (N = 7) and early blind (N = 7) age-matched participants (non-parametric Mann-Whitney test suggested no significant difference between groups on age: W = 2150, p-value = 0.21). We verified the effect of the group in this subset of data, obtaining the same significantly lower performance of the early blind than the blindfolded sighted control group (*χ*^*2*^(1) = 28.8; *p-value* < 0.001; see Fig. 4).

Previous literature found that a prolonged period of blindness could impact neural circuits for spatial representations in the auditory domain (Amadeo et al., 2019), but not in the haptic domain (Bertonati et al., 2020; Palacios et al., 2023). We tested the potential effect of the duration of blindness on haptic cross-sectioning. We selected late blind participants and conducted an exploratory mixed-effects logistic regression model, considering accuracy as the outcome and the duration of blindness expressed in years and computed by subtracting the onset of blindness from current age at testing (mean of duration of blindness (SD): 21.8 (12); range: 11–50 years) as a continuous predictor, with a random intercept for each participant:

$$\mathrm{accuracy}\;\sim\;\mathrm{duration}\;\mathrm{of}\;\mathrm{blindness}\;+\;\left(1\left|\mathrm{participant}\right.\right)$$

**Fig. 5:**
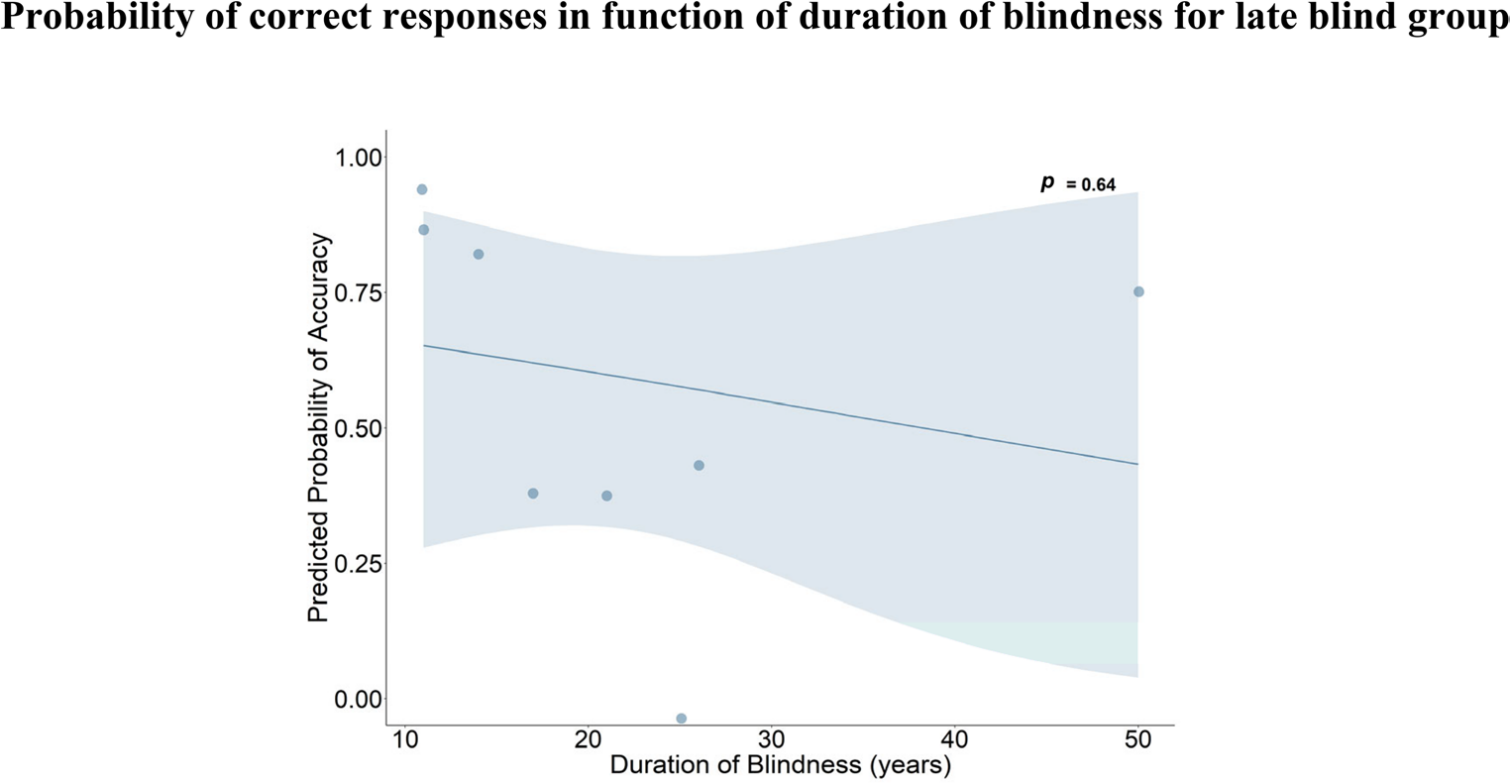
The predicted probability for correct responses plotted in function of the duration of blindness expressed in years for late blind individuals. There is no significant relationship between the two (*p*-value =0.64)

No significant effect of the duration of blindness was found (*β* = −0.023, standard error SE = 0.05, *z* = −0.46, *p-value* = 0.642; odds ratio = 0.98; see Fig. 5).

## Discussion

Haptic exploration is an important daily life activity. The present study aimed at investigating the effect of visual impairments on haptic exploration in a cross-sectioning task. To respond to this scientific question, we 3D printed a selected subset of the visual Santa Barbara Solid Test (SBST) and asked blindfolded sighted, low vision, late, and early blind participants to haptically explore and recognize the 2D section of the sectioned 3D solids. As mentioned previously in the manuscript, we defined the thin response options as “2D sections” although these are physically thin 3D objects that can be explored haptically, to highlight their geometric definition as planar cross-sections and to mirror the visual version of the task. Given the proposed role of early visual experience in calibrating spatial representations, we hypothesize that individuals who are early blind would exhibit reduced performance in precise orientation discrimination and complex spatial transformations.

Our results supported this hypothesis. Early blind participants revealed a substantially lower accuracy in comprehending the complex spatial features of the 3D shapes. Considering the mixed findings in literature, our result is in line with some previous studies, suggesting that the lack of vision in the crucial first stages of life prevents individuals from forming accurate and reliable spatial representations in the remaining senses (i.e., touch). The cross-sensory calibration theory (Burr & Gori, 2012; Gori, 2015) states that vision, which is the most accurate sense in spatial perception, calibrates the other senses to reach optimal spatial processing. In Bertonati et al. (2020), early blind participants showed substantially lower percentage of correct responses than late blind and blindfolded sighted participants in an haptic version of the Kohs Block Design Test. The authors attribute this result to the complex requirement to manipulate simultaneously haptic information generating multiple spatial representations. Similarly, Vecchi and colleagues (2004) showed that congenital blindness affected the ability to retain multiple spatial information simultaneously, while leaving preserved sequential sensory processing. In the present work, the simultaneous exploration of the whole solid and section plane while considering their spatial relationship at the same time is a fundamental requirement for succeeding.

As mentioned in the Introduction, a successful cross-sectioning trial involves different stages: (i) building a coherent representation of the 3D object; (ii) transforming this representation, taking into account the relationship between the section and the solid; (iii) keeping in memory the resulting 2D cross-sectional shape. This last step requires working memory, which is a brain system that provides temporary storage and manipulation of the information (Baddeley, 1992). Possible explanations for the observed group differences and their theoretical implications are discussed in the following paragraphs.

We propose that the major cause of early blind poorer performance reflects a greater difficulty in transforming the mental representation of the solid collected through touch and use it in relationship with the section (stage ii). This transformation requires not only constructing an accurate representation through touch, but also mentally manipulating that representation to infer its internal spatial structure. As previously mentioned, early blind individuals often show reduced efficiency to process multiple sensory cues simultaneously (Bertonati et al., 2020; Vecchi et al., 2004). Previous research demonstrates that congenitally blind participants significantly differ in the strategy applied for haptic object recognition, with more simultaneous touches and thus less exploration time (Davidson et al., 1974; Leo et al., 2022). Although we did not directly investigate specific exploration strategies of each population, it is plausible that such differences contributed to the observed group effects. Indeed, simultaneous and fast exploration of shapes may lead to a poorer comprehension of the complex spatial relationship between the section and the 3D solid, which could require a more parallel exploration.

Another task that investigates mental object transformation is the mental rotation test (Peters et al., 1995; Shepard & Metzler, 1971; Vandenberg & Kuse, 1978), which works with stable, predefined shape features. Typically, results show that the amount of rotation required to determine whether two objects are similar strongly correlates with the response time of participants (Peters et al., 1995; Shepard & Metzler, 1971; Vandenberg & Kuse, 1978). In other words, the greater the rotation, the longer the time of response. Interestingly, this result concerns different sensory domains: it is true for visual mental rotation, but also for haptic mental rotation (Robert & Chevrier, 2003; Sveistrup et al., 2023; Tivadar et al., 2019). In addition, rotation amplitude of haptically-rendered objects affects mental rotation in visually impaired people as well (Tivadar et al., 2020). Differently from the MRT, the cross-sectioning task challenges participants to mentally “slice” a 3D object and integrate these cross-sections to understand the whole shape, subtending similar but different spatial abilities (Cohen & Hegarty, 2012; Ratliff et al., 2010). As the authors of the SBST mentioned (Cohen & Hegarty, 2012), “spatial visualization ability is not the sole determinant of performance on the Santa Barbara Solids Test. Rather, this test measures a skill, […] which is somewhat distinct from those measured by the Vandenberg Mental Rotation Test.” (p. 871). In light of the found group differences, our results support the involvement of spatial processes specific to cross-sectional reasoning, which appear to differ from those underlying mental rotation, even when performed haptically.

Previous studies report that congenitally or early-blind individuals show systematic biases in reproducing and recognizing familiar object shapes compared to their sighted peers (Tian et al., 2024), suggesting that mental representation of objects acquired through touch is impaired without visual experience. However, this conclusion is not unequivocally supported by the literature. Other research shows that early-blind individuals can exhibit even enhanced tactile discrimination and shape recognition than sighted and late-blind individuals (Norman & Bartholomew, 2011; Occelli et al., 2016) or equal performance (Leo et al., 2022). Taken together, these findings suggest that early blindness does not necessarily entail a generalized deficit in object recognition or in the coherent mental representation of three-dimensional shapes. Thus, we argue that the poorer performance observed in the present study is unlikely to stem from impaired 3D object recognition per se (the abovementioned stage i). Rather, it more plausibly reflects difficulties at a later processing stage, i.e., in transforming and linking the 3D representation to its corresponding sectional view.

Partially, different working memory (Baddeley, 1992) functioning may subtend the early blind performance. In our task, even though working memory was not explicitly measured, participants were required to retain the spatial configuration of the sectioned solid through the exploration of the four possible answers (stage iii). Mixed results are found concerning potential differences between blind groups’ and sighted working memory, especially considering verbal and spatial tasks together (see the systematic review: Sepúlveda-Palomo et al., 2024). However, previous research suggests that congenitally blind individuals exhibit comparable working memory performance to sighted individuals in tasks involving the exploration and re-execution of a spatial sequence through touch (Ruggiero & Iachini, 2010). This implies that our findings are unlikely to be attributed to working memory impairments.

Another weaker explanation of our findings strictly linked to strategies of exploration is sensorimotor ability. In our task, it is essential to combine information coming from the sense of touch and coordinate the hand movements relative to the object (Gori et al., 2024; Sciutti & Sandini, 2022). Overall, blind people show similar hand movements to their sighted peers (e.g., Castiello et al., 1983). However, some studies reported that blind individuals have distorted mental representations of their hands (Coelho et al., 2024; Rakesh Kottu & Lazar, 2025) that may translate to poorer coordination. However, these distortions have been observed in both early and late blind groups (Coelho et al., 2024; Rakesh Kottu & Lazar, 2025), suggesting that hand representation may not account for our observed results.

One could also attribute early blind group results to a lack of early experience with geometric concepts and exploration. Late blind and low vision participants had some visual experience with solids and geometry, likely since primary school. Although our 3D objects consisted of simple geometric solids to minimize the influence of familiarity on this task, we cannot exclude that previous general visual experience with geometrical shapes and educational background could have helped their haptic exploration strategies. However, young children aged 5–10 years old demonstrated to benefit from visual-haptic exploration of solids to correctly depict the 2D section (Gori et al., 2024), suggesting that vision plays an important role in guiding haptic spatial representation in early developmental stages, regardless of practical experiences with shapes. This means that it is more likely that the lack of visual calibration in the early stages of life is responsible for the less efficient haptic processing. Cross-sensory calibration theory (Burr & Gori, 2012; Gori, 2015) for sophisticated spatial tasks such as cross-sectioning seems to be the more compelling framework to explain the present findings.

The same interpretation can explain the absence of differences in the other three groups as well, the late blind and low vision participants, and the sighted controls. Indeed, all three groups, regardless of current visual acuity, have an early visual experience that guarantees the visual calibration over the haptic sense. Notably, the youngest participant classified as “late blind” became blind at 6 years of age: this, in line with previous literature, indicates that the visual calibration already occurred before school age, and therefore, even before any systematic experience with geometrical problems. In addition, we observed that the duration of blindness, spanning from 11 to 50 years, did not influence haptic performance. This may suggest that the ability to recognize the 2D shapes of 3D sectioned solids was not modulated by the time lived without visual information. However, due to the very small sample of late blind participants (*N* = 8), our study was underpowered to detect potential effects of long-term blindness on haptic spatial abilities, and the observed non-significant result should be interpreted with caution and not be interpreted as evidence of absence. Nonetheless, in line with previous findings (Bertonati et al., 2020; Palacios et al., 2023), it seems plausible that spatial reasoning is greatly affected by the lack of visual experience during the first years of life, rather than by prolonged absence of vision.

We found no effect of trial complexity on performance for any groups. In the original Santa Barbara Solid Test (Cohen & Hegarty, 2007), the authors presented 30 trials that could be classified according to the mentioned definition: 5/30 are low level of difficulty (simple shapes with orthogonal section); 5/30 are medium level of difficulty (simple shapes with oblique section); the remaining trials are high level of difficulty, with embedded or joined 3D shapes with orthogonal or oblique sections. For our experiment, we decided to select a subset of those trials to minimize participants’ effort. At the same time, we selected more trials from the medium level of difficulty to reduce potential frustration, especially for the clinical populations involved.

We originally hypothesized that oblique cutting would introduce greater difficulty in interpreting the proportion of the resulting cross-sectional shape, while we expected that embedded and joined solids would increase the number of spatial reasoning steps to find the correct section, as reported for the visual version of the test (Cohen & Hegarty, 2007). However, in light of our interpretation of the group effect within the cross-calibration theory, it is plausible that even the simplest condition had challenged the early blind participants in their ability to accurately perceive and identify the correct section. This suggests that the cognitive demands of the task may have been more complex than initially anticipated, even for conditions that were designed to be easier. Importantly, difficulty level classification was based on the original visual version of the task. It is therefore questionable whether this categorization can be directly applied to the haptic modality. It is likely that haptic complexity may rely on different spatial features of the cross-sectioned solids. This could explain why, numerically, the medium-level led to lower accuracy percentage (see Fig. 5), especially for low vision and sighted groups.

## Future studies

Future studies should replicate these findings with larger and more balanced samples, include more varied trials, and explore exploration strategies to better understand the mechanisms underlying haptic cross-sectioning performance. Moreover, even previous research did not find any temporal influence on haptic performance (Bertonati et al., 2020), future works could investigate both exploration and response time to provide further insights into exploration strategies. A proper balance design would help disentangle the specific processing stage and cognitive mechanisms underlying the found group differences. In addition, it will be important to consider both low vision people who experienced visual impairment early versus later in life, to better disentangle the influence of early vision on haptic cross-sectioning. Importantly, future investigations may examine the optimal strategies for successful performance in recognizing haptic cross-sections and why congenitally blind modus operandi is not the most apt to achieve this task.

Future research could also focus on uncovering the neural mechanisms that explain why the re-organization observed in occipital cortices of blind individuals (Amedi et al., 2010; Xu et al., 2023) is insufficient to successfully perform the present task. Additionally, employing complementary or control tasks could help delineate the specific contributions of each cognitive process, building on the foundational evidence provided here.

## Limitations

As briefly mentioned, this study has some limitations that should be considered. The small and unbalanced sample size across groups, along with the difference in mean age, limits the statistical power and generalizability of our results. In addition, the lack of detailed information for the small low vision group prevents us from drawing broader conclusions about this population. Further investigation with a larger sample size and more varied trial conditions could help clarify these findings and potentially reveal subtler effects that were not detectable with the current experimental setup. However, simulation-based sensitivity analysis indicates that the observed group effect, i.e., the substantially lower accuracy of early blind participants, is reliably detectable despite the modest sample. In contrast, smaller or more subtle effects, such as trial difficulty, interaction, or the duration of blindness in the late blind group (*N* = 8), could not be reliably detected and should be interpreted with caution. In addition, our study was not adequately powered to test for potential sex effects or sex-by-group interactions. Given the relatively small sample sizes within each visual status group, including sex as an additional factor would have resulted in unstable parameter estimates. Previous research indeed suggests that sex differences in spatial abilities may vary according to tasks and task definition (Bartlett & Camba, 2023; Linn & Petersen, 1985; Voyer et al., 1995). Evidence regarding such differences in cross-sectioning tasks remains mixed (Cohen & Hegarty, 2012; Neigel et al., 2017), highlighting the need for future research.

## Conclusions

With the present study, we found that early visual impairment leads to reduced performance in a haptic cross-sectioning task (*p-value* < 0.001); besides, variations in task difficulty did not affect performance (*p-value* = 0.22). Overall, our findings provide new insights into how vision loss shapes individuals’ ability to perceive and understand complex spatial information. It contributes to the broader understanding of sensory processing in visually impaired populations. The study’s findings may have significant implications for educational strategies and assistive technologies. Since early vision loss is found to limit spatial reasoning in tasks that require cross-sectioning, this informs the development of new tools or training programs that help blind individuals better visualize complex spatial features, which is also significant for common daily activities.

## Supplementary Information

Below is the link to the electronic supplementary material.

Supplementary Material 1 (DOCX 3.01 MB)

## Data Availability

All raw data will be publicly available on the Zenodo repository (https://zenodo.org/record/20050432). The present study has not been pre-registered.
