# Supplementary material for: Early visual experience influences haptic cross-sectioning ability
Source: Psychol Res. 2026 May 22;90(3):98. doi: 10.1007/s00426-026-02310-9 (PMC13197313; doi:10.1007/s00426-026-02310-9)

# Early visual experience influences haptic cross-sectioning ability

**Psychological Research**

Monica Gori^1,2^, Margherita Di Gaudio^1,3^, Diego Torazza^4^, Silvia Zanchi^1*^

^1^ Unit of Visually Impaired People, Istituto Italiano di Tecnologia, Genoa, Italy

^2^IHMC, Institute for Human & Machine Cognition, 40 South Alcaniz St. Pensacola, FL 32502, USA

^3^DIBRIS Department, Università di Genova, Genoa, Italy

^4^Generative Bionics S.R.L., Genoa, Italy

*Corresponding: silvia.zanchi@iit.it

# Supplementary Information

Figure S1. The top panel shows the familiarization trial solid (the sphere, on the left) alongside the 10 experimental solids arranged on a table. The bottom panel displays also all the available cross-section options.


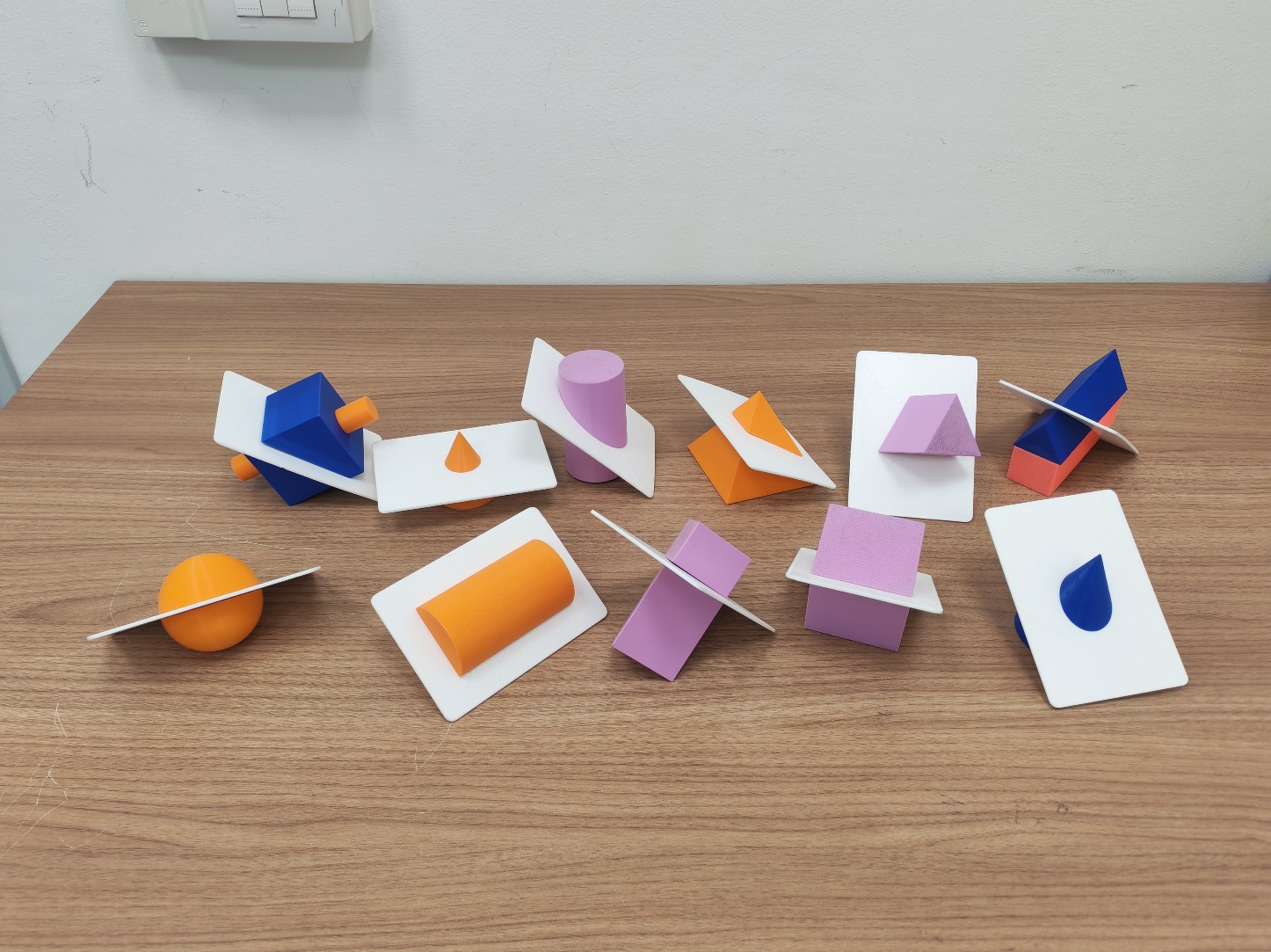

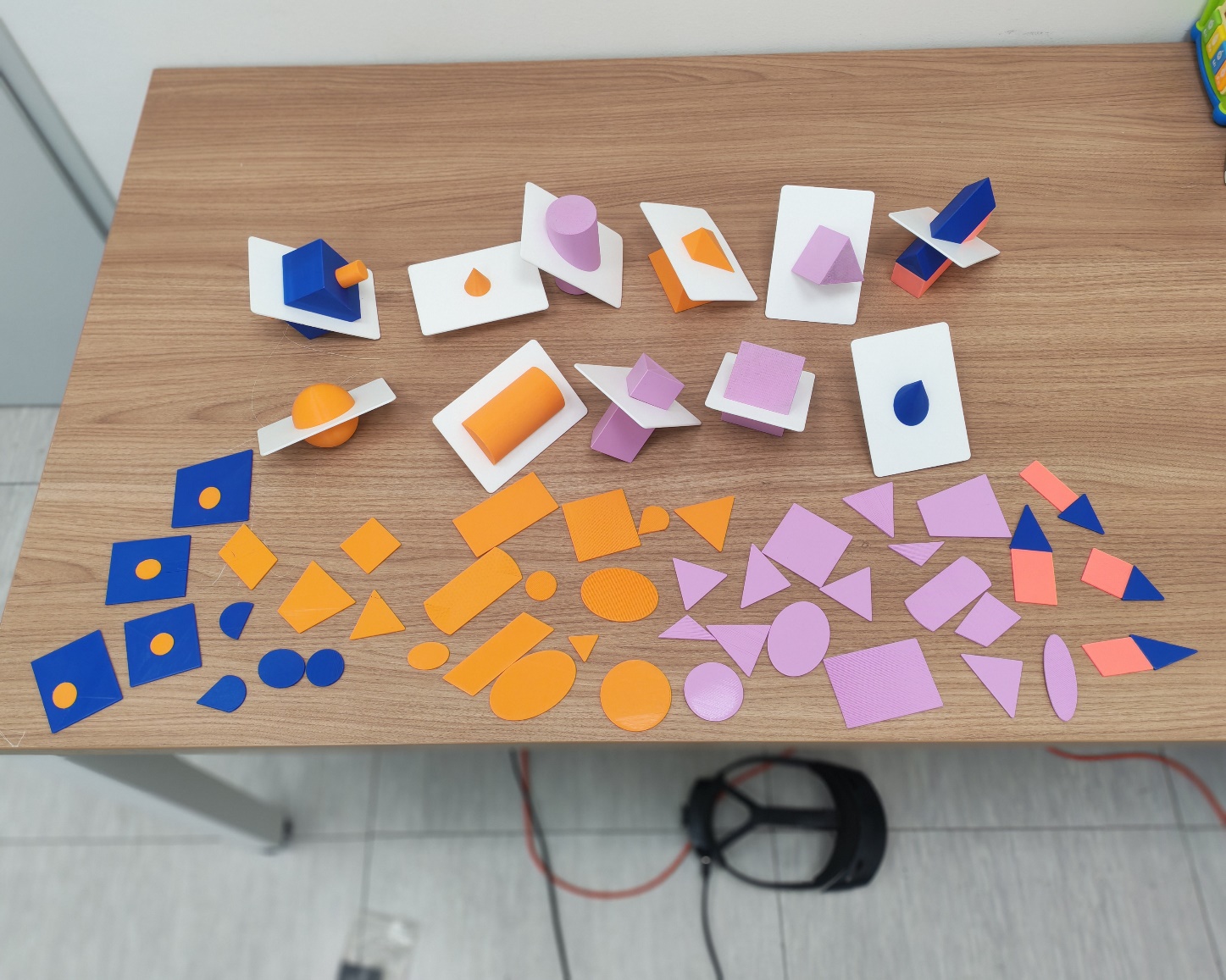

Supplement: Supplementary file 1 — Supplementary Material 1 (DOCX 3.01 MB) [file 426_2026_2310_MOESM1_ESM.docx]
